# Metabolomics comparison of metabolites and functional pathways in the SH-SY5Y cell model of Parkinson's disease under PEMF exposure

**DOI:** 10.1016/j.heliyon.2024.e26540

**Published:** 2024-02-16

**Authors:** Li-na Zhu, Deng Chen, Chengqi He

**Affiliations:** a Department of Rehabilitation Medicine, Key Laboratory of Rehabilitation Medicine, Institute of Rehabilitation Medicine, West China Hospital, Sichuan University, Chengdu, 610041, Sichuan, China; b Department of Neurology, West China Hospital, Sichuan University, Wai Nan Guo Xue Lane 37 #, Chengdu, 610041, Sichuan, China

**Keywords:** PEMF, Metabolomics, PD, SH-SY5Y, Cell model

## Abstract

**Objective:**

PEMF is an emerging technique in the treatment of Parkinson's disease (PD) due to its potential improvement of movement speed. The aim of this study was to investigate the metabolic profiles of pulsed electromagnetic fields (PEMFs) in an SH-SY5Y cell model of PD.

**Methods:**

The SH-SY5Y cell model of PD was induced by 1-methyl-4-phenylpyridinium (MPP^+^). Liquid chromatography mass spectrometry (LC‒MS)-based untargeted metabolomics was performed to examine changes in the PD cell model with or without PEMF exposure. We conducted KEGG pathway enrichment analysis to explore the potentially related pathways of the differentially expressed metabolites.

**Results:**

A total of 275 metabolites were annotated, and 27 significantly different metabolites were found between the PEMF treatment and control groups (VIP >1, *P* < 0.05), mainly including 4 amino acids and peptides, 4 fatty acid esters, 2 glycerophosphoethanolamines, 2 ceramides and 2 monoradylglycerols; among them, 12 metabolites were upregulated, and 15 were downregulated. The increased expression levels of glutamine, adenosine monophosphate and taurine were highly associated with PEMF stimulation in the PD model. The enrichment results of differentially abundant metabolite functional pathways showed that biological processes such as the mTOR signaling pathway, PI3K-Akt signaling pathway, and cAMP signaling pathway were significantly affected.

**Conclusion:**

PEMFs affected glutamine, adenosine monophosphate and taurine as well as their functional pathways in an in vitro model of PD. Further functional studies regarding the biological effect of these changes are required to evaluate the clinical efficacy and safety of PEMF treatment in PD.

## 1 Introduction

Parkinson's disease (PD) is one of the most common neurodegenerative diseases [1], affecting millions of people worldwide and imposing a great economic burden [2,3]. Perplexing causes (i.e., age, genes and environment) are involved in the development and progression of PD [4,5], among which the severity and duration of PD have a relationship with the loss of dopaminergic (DA) neurons from the substantia nigra pars compacta (SNpc) [6]. However, the underlying mechanism of the selective degeneration of dopaminergic neurons is unclear, and thus, measures to effectively protect them have not been established.

The primary therapeutic method for PD is still symptomatic pharmacologic treatment, the efficacy of which decreases over time [7]. There are also many limitations in applying pharmacologic treatment. Levodopa and dopamine agonists are not effective in controlling nonmotor symptoms (NMS). Adverse events (AEs) and poor medical adherence are also problematic. Above all, long-term treatment with levodopa is associated with the emergence of motor complications, particularly fluctuations and dyskinesias. These issues could strongly impact quality of life (QOL) in the long run. Thus, developing innovative treatments, especially other new nonpharmacological treatments for PD, is urgently needed.

Electromagnetic stimulation is a noninvasive and nonpharmacological physiotherapy currently under research for PD treatment. On the one hand, evidence has shown that a pulsed electromagnetic field (PEMF) can enhance cellular activity and stimulate growth-related responses and regeneration [8,9]. Additionally, even though the effect has not been confirmed to be attributed to PEMF, Jeasen et al.’s study suggested that PEMF therapy might potentially exert neuroprotective effects by promoting neural repair and/or protecting dopaminergic neurons, thus improving motor performance [10]; however, studies regarding the effect of an extremely low-frequency electromagnetic field (ELF-EMF), a similar form of PEMF, on some in vitro models of PD indicated otherwise. A study reported that ELF-EMF altered the sensitivity of SH-SY5Y cells in response to a PD model-inducing reagent by altering redox homeostasis and thiol content [11]. These paradoxical findings warrant further investigation.

Metabolomics is a rapidly emerging technique that has high sensitivity to biochemical changes and could be utilized to identify crucial biomarkers and pathways by profiling metabolite changes. Many studies have been dedicated to exploring metabolic dysregulations in relation to PD prognosis and diagnosis-based liquid chromatography‒mass spectrometry (LC‒MS) by using a number of biofluids, including blood, saliva, urine and cerebrospinal fluid (CSF), and several potential metabolic biomarkers of PD have been reported [5].

Lewitt et al. demonstrated that the alteration in N-acetylation of amino acids in the CSF appeared to be related to excitotoxicity and oxidative stress in the pathogenesis of PD [12]. Previous studies have also indicated that several amino acids (e.g., tryptophan, tyrosine) [13], acylcarnitines [14], quinolinic acid (QA)/kynurenic acid (KA) ratio [15], N8-acetyl spermidine and lipids [5,16] were altered in the plasma of PD patients. These metabolites might be potential biomarkers or novel therapeutic targets for studying the physiology and pathology of PD.

In this study, we aimed to evaluate the effect of PEMFs on a cell model of PD by using an LC‒MS-based metabolomics approach, which could provide insights into their biological function and enlighten further mechanistic studies.

## 2 Materials and methods

### 2.1 Cell culture and MPP ^+^ induction

The human neuroblastoma SH-SY5Y cell line was purchased from iCell Bioscience Inc. (Shang Hai, China) and was derived from the American Type Culture Collection (ATCC, Cat# CRL‐2266). The cells were cultured on 10 cm diameter plates in Nutrient Mixture Ham's F-12 (DMEM/F12, 1:1) medium supplemented with 10% fetal bovine serum without antibiotics and maintained at 37 °C in a 5% CO2 incubator. Cells were incubated with 1-methyl-4-phenylpyridinium (MPP^+^) at 500 μM at 37 °C when confluency was between 60 and 70%. After 36 h of incubation, we replaced the medium with fresh medium.

### 2.2 PEMF stimulation

**Fig. 1:**
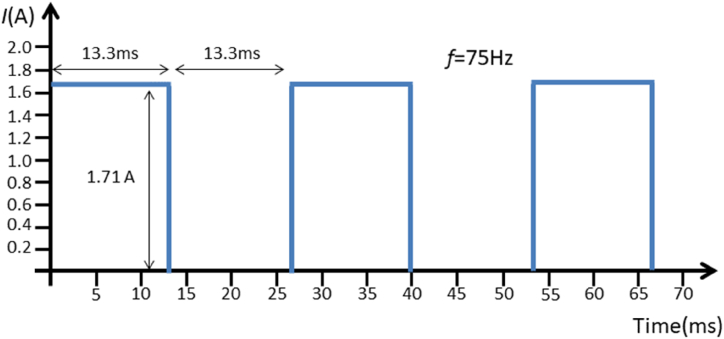
The PEMF waveform characteristics in this study.

The experimental setup of our PEMF exposure device consisted of four parts: a tunable switching power supply unit, a pulse generator, signal amplification, and a circular Helmholtz coil (School of Manufacturing Science and Engineering, Sichuan University, Chengdu, China). PEMF stimulation parameters were as follows: signal waveform, square wave; intensity, 3 mT; frequency, 75 Hz; duty ratio, 50% (Fig. 1). The magnetic field intensity was measured by a hand‐held Gauss meter (HT201; Hengtong, Shanghai, China). The quantities of cells were tested using a Countstar™ Altair cell counter before PEMF stimulation. The PEMF intervention group (6 plates) had a daily 60 min PEMF therapy session (75 Hz, 3 mT) for 3 days. The PEMF coil device was placed inside a cell culture incubator with a humidified environment at 37 °C. The cells in the control group (other 6 plates) were housed in the same incubator at the same time, but the magnetic field was shielded by a metal shell around the coil. Then, cells were collected after completing all interventions.

### 2.3 Biospecimen collection and processing

We adopted the following protocol for the LC/MS untargeted metabolomics study: cells were collected with a scraper, resuspended in PBS, and then accurately counted under a microscope. Subsequently, cell samples were transferred into centrifuge tubes at 800*g for 5 min. After discarding the supernatant, samples were immediately frozen in liquid nitrogen and stored at − 80 °C for later use.

Before metabolomics analysis, the samples were ground in a centrifuge tube with grinding beads and then deproteinized with methanol/water (1/3) containing internal standards. After further grinding with a frozen tissue grinder for 6 min (-10 °C, 50 Hz) and low-temperature ultrasonic extraction for 30 min (5 °C, 40 kHz), the samples were then left at −20 °C for 30 min. In addition, the samples were centrifuged at 13000×*g* for 10 min at 4 °C, and the supernatant was taken for mass spectrometry measurement.

### 2.4 LC‒MS analysis

Ten microliter aliquots of the samples were injected into a Thermo Scientific UHPLC equipped with a column (ACQUITY UPLC HSS T3, 1.8 μm, 100 mm × 2.1 mm; Waters, Milford, USA). Chromatography was conducted at 40 °C during the analysis. Solvent A was 95% water, 5% acetonitrile and 0.1% formic acid. Solvent B included 47.5% acetonitrile, 47.5% isopropyl alcohol, 5% water and 0.1% formic acid. The flow rate was set at 0.4 ml/min. The solvent gradient for sample analysis was as follows: 0 min, 100% solvent A; 0.1 min, 95% solvent A; 2 min, 75% solvent A; 9 min, 100% solvent B; 13 min, 100% solvent B; 13.1 min, 100% solvent A; 16 min, 100% solvent A.

MS was performed on Thermo Scientific Q-Exactive mass spectrometer equipped with an electrospray ionzation (ESI) source. The mass scan was performed in the range of 70 *m*/*z* to 1075 *m*/*z*. Positive and negative modes were used to obtain data by optimizing the parameters: Sheath gas flow rate, 40 arb; Aux gas flow rate, 10 arb; Heater temperature, 400 °C; Capillary temperature, 320 °C; Spray voltage (+) 3500V; Spray voltage (−) −2800V.

### 2.5 Quality control

To assess the repeatability of sample processing and the stability of the instrument, quality control (QC) samples were prepared by mixing equal volumes (10 μl) of each sample, which were tested in the same way as the analytical sample.

### 2.6 Pattern recognition and pathway analysis

The metabolomics processing software ProgenesisQi 2.3 (Waters Corporation, Milford, USA) was used to process the raw data, including baseline filtering, retention time correction, peak identification and alignment. Subsequently, a data matrix of information was generated, including the retention time (RT), mass-to-charge ratio (*m*/*z*) and peak intensity. For the identification of metabolic molecules, the available databases were used: the Human Metabolome database (HMDB) (http://www.hmdb.ca/) and the Metlin database (https://metlin.scripps.edu/). The metabolic pathways involved in the discrepant metabolites were revealed by the Kyoto Encyclopedia of Genes and Genomes (KEGG) pathway.

### 2.7 Statistical analysis

All multivariate statistical analyses were performed using the Majorbio Cloud Platform (www.majorbio.com). To compare metabolite profiles between the PEMF group and the control, principal component analysis (PCA), orthogonal partial least squares (OPLS-DA) analysis and partial least squares discriminant analysis (PLS-DA) were conducted in our study. Variable importance in the projection (VIP >1) combined with a T test (Student's test, *P* < 0.05) were used to detect the discrepant metabolites. In KEGG enrichment analysis, scipy.stats (Python packages) (https://docs.scipy.org/doc/scipy/) was performed to identify the significantly enriched pathways by Fisher's exact test, and *P* < 0.05 was selected as statistically significant.

## 3 Results

### 3.1 Reliability of measurements

In this study, we employed 6 plates of cells in the interventional group with PEMF stimulation and another 6 plates in the control group. The metabolic patterns of cell samples from the PEMF intervention group and control group were analyzed. The representative positive (POS) and negative (NEG) ion base peak intensity chromatograms of the QC samples showed a relatively uniform distribution throughout the experimental period, suggesting good stability of the MS system. These results are available in the supplementary material.

**Fig. 2:**
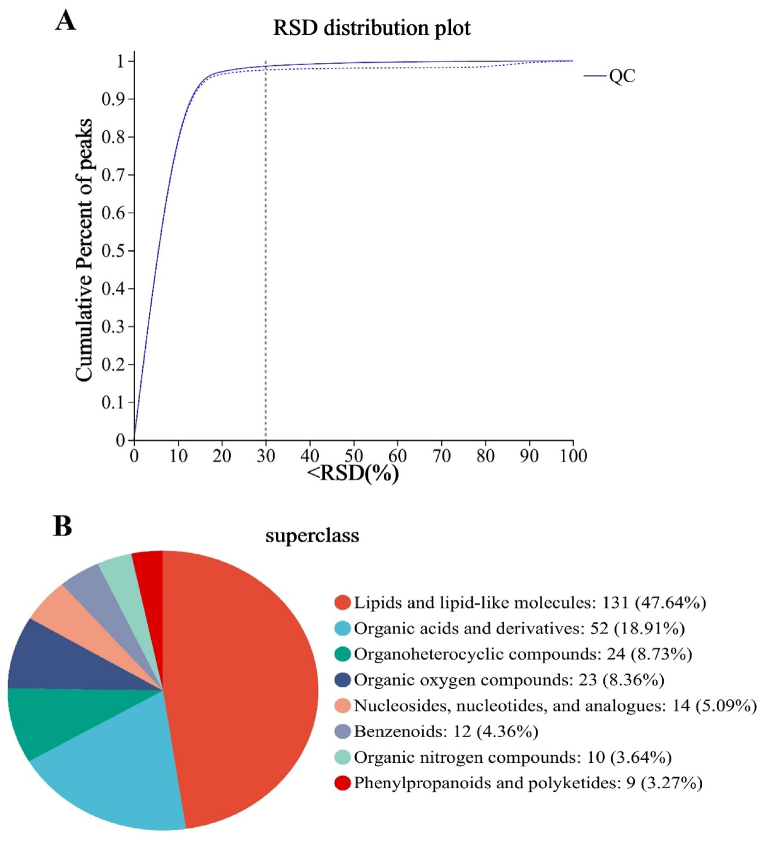
(A) Relative standard deviation (RSD) distribution of quality control samples; (B) Compound classification in the Human Metabolome Database (HMDB).

As shown in Fig. 2A, more than 90% of the relative standard deviation (RSD) values of the QC samples were below 30%, indicating that the data were generally qualified. Moreover, the reproducibility of the QC samples was confirmed by the PCA scores, and no sample was beyond Hotelling's t-squared confidence circle (95%).

### 3.2 Metabolite annotation

After preprocessing, 5158 and 4529 effective peaks were detected in the POS and NEG scan modes, respectively. Based on the metabolome databases, a total of 275 metabolites were annotated in the HMDB (Fig. 2B), which were divided into 131 lipids and lipid-like molecules, 52 organic acids and derivatives, 24 organoheterocyclic compounds, 23 organic oxygen compounds, 14 nucleosides and analogs, 12 benzenoids, 10 organic nitrogen compounds and 9 phenylpropanoids and polyketides; among them, lipids and lipid-like molecules occupied the highest proportion, accounting for 47.64%.

The mass spectrum information was also imported into the KEGG pathways for matching. We found that the KEGG pathways were mainly amino acid metabolism (arginine biosynthesis, arginine and proline metabolism, phenylalanine metabolism, alanine, aspartate and glutamate metabolism), carbohydrate metabolism (glyoxylate and dicarboxylate metabolism, TCA cycle), lipid metabolism (glycerophospholipid metabolism), membrane transport (ABC transporters), neurodegenerative disease (pathways of neurodegeneration-multiple diseases, Parkinson disease) and nucleotide metabolism (purine metabolism). The top 20 KEGG pathways are shown in Table 1.

**Table 1:** Top 20 KEGG pathway.

Pathway	Pathway ID	Number
Purine metabolism	map00230	12
ABC transporters	map02010	11
Central carbon metabolism in cancer	map05230	10
Alanine, aspartate and glutamate metabolism	map00250	8
Glycerophospholipid metabolism	map00564	8
Glyoxylate and dicarboxylate metabolism	map00630	7
Arginine biosynthesis	map00220	7
Arginine and proline metabolism	map00330	7
Neuroactive ligand-receptor interaction	map04080	7
Protein digestion and absorption	map04974	7
Pathways of neurodegeneration - multiple	map05022	6
Glutathione metabolism	map00480	6
Sphingolipid signaling pathway	map04071	6
Taste transduction	map04742	6
Diabetic cardiomyopathy	map05415	6
Phenylalanine metabolism	map00360	5
Citrate cycle (TCA cycle)	map00020	5
Glucagon signaling pathway	map04922	5
Aminoacyl-tRNA biosynthesis	map00970	5
Parkinson disease	map05012	5

Notes: Top 20 KEGG pathways to which the annotated metabolites belong.

### 3.3 Multivariate statistical analysis results

**Fig. 3:**
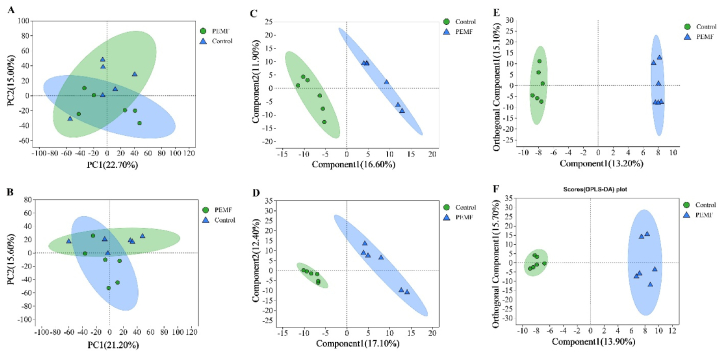
Multivariate statistical analysis in the PEMF group and PD group. Principal component analysis (PCA) score chart in positive (A) and negative (B) modes. Partial least squares discriminant analysis (PLS-DA) score chart in positive (C) and negative (D) modes. Orthogonal partial least squares discriminant analysis (OPLS-DA) score chart in positive (E) and negative (F) modes.

The PCA score chart in positive and negative modes tended to separate the PEMF group and PD group (Fig. 3A and B), but there were cross-distributed areas. PLS-DA (Fig. 3C and D) and OPLS-DA models (Fig. 3E and F) were performed to reveal the general metabolic changes. These analyses achieved a better presentation of differences with a clear separation. These model parameters in both positive and negative ion modes are shown in Table 2. The values of R2Y (cumulative) and Q2Y (cumulative) indicate that these models had relatively high reliability and predictability.

**Table 2:** Model parameters of multivariate statistical analysis on POS and NEG.

	OPLS-DA		PLS-DA	
	R2Y (cum)	Q2Y (cum)	R2Y (cum)	Q2Y (cum)
POS	0.998	0.488	0.998	0.748
NEG	0.99	0.554	0.99	0.704

OPLS-DA, orthogonal partial least squares analysis; PLS-DA, partial least squares discriminant analysis; POS, positive mode; NEG, negative mode; and cum, cumulative.

Response permutation testing (RPT) was then conducted to assess whether the OPLS-DA and PLS-DA models were accurate. For the PLS-DA model, R2 and Q2 in RPT were 0.9975 and 0.4815 in POS ion mode and 0.9764 and 0.2093 in NEG ion mode, respectively. For the OPLS-DA model, R2 and Q2 in RPT were 0.9977 and 0.1025 between the PEMF and PD groups (POS) and 0.9788 and 0.0772 between the PEMF and PD groups (NEG), respectively. The details are shown in the supplementary material. These results further revealed that these two models were established successfully and gave good stability for subsequent analysis.

### 3.4 Differentially abundant metabolite analysis

**Fig. 4:**
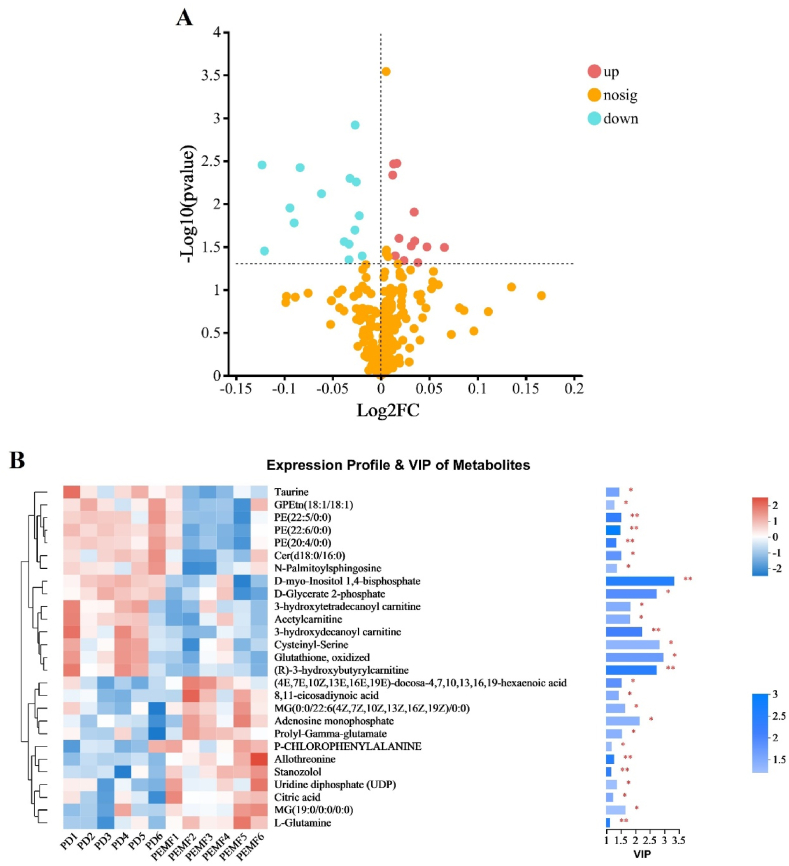
(A) Volcano plots of the identical metabolites. Blue and purple dots indicate upregulated and downregulated metabolites, respectively. Red dots mean no difference between control and PEMF intervention group; (B) Hierarchical cluster dendrogram of significantly different metabolites. ‘red’ indicates high relative expression, and ‘blue’ indicates low relative expression. ‘PD’ and ‘PEMF’ respectively represent control group and PEMF intervention group (each group with six samples). Levels of significance are defined as *P < 0.05, **P < 0.01. (For interpretation of the references to color in this figure legend, the reader is referred to the Web version of this article.)

A total of 27 metabolites that were significantly different between these two groups were found (VIP >1, P < 0.05). Among them, 12 metabolites were upregulated, and 15 were downregulated. The upregulated glutamines included l-glutamine (VIP = 1.12, *P* = 0.003) and prolyl-gamma-glutamate (VIP = 1.53, *P* = 0.03). Also, the adenosine monophosphate was upregulated (VIP = 2.14, *P* = 0.03). The downregulated fatty acid esters (also categorized as lipids and lipid-like molecules) were 3-hydroxydecanoyl carnitine (VIP = 2.23, *P* = 0.008), 3-hydroxytetradecanoyl carnitine (VIP = 1.82, *P* = 0.03), (R)-3-hydroxybutyrylcarnitine (VIP = 2.73, *P* = 0.004) and acetylcarnitine (VIP = 1.80, *P* = 0.03). In the downregulated organic oxygen compounds, there were cysteinyl-serine (VIP = 2.82, *P* = 0.04) and oxidized glutathione (VIP = 2.95, *P* = 0.02). Volcano plots and hierarchical clustering analysis were used to depict the expression levels of these notably different metabolites between the two groups, which are shown in Fig. 4A and B, respectively.

In the KEGG analyses, 10 metabolites with significant differences were annotated and involved 64 different biological pathways, which are listed in the supplementary material.

**Fig. 5:**
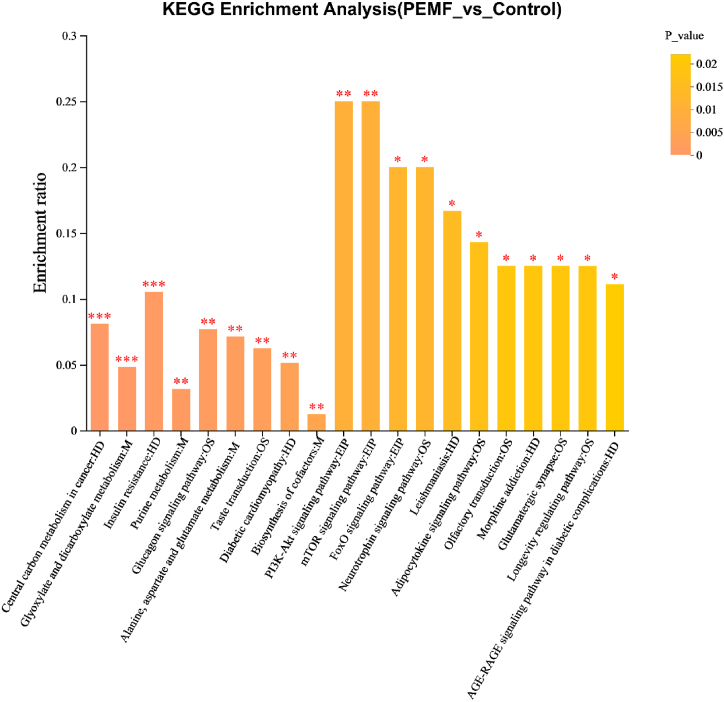
KEGG pathway enrichment of significantly different metabolites between the control and PEMF intervention group. The darker the default color, the more significant the enrichment of the KEGG term. Levels of significance are defined as *P < 0.05, **P < 0.01, and ***P < 0.001. (For interpretation of the references to color in this figure legend, the reader is referred to the Web version of this article.)

The pathway enrichment analysis identified key pathways, including the mTOR signaling pathway, PI3K-Akt signaling pathway, FoxO signaling pathway, neurotrophin signaling pathway, and glutamatergic synapse. The details are displayed in Fig. 5.

## 4 Discussion

In this comparison, we used 12 plates of cells and divided them into 2 groups in a 1:1 ratio to perform a metabolomics study. Our study included a direct comparison regarding the effect of PEMFs on the MPP + -treated SH-SY5Y cell model of PD. According to a review, over 200 studies used an MPP + -treated SH-SY5Y cell model, thus making it the most frequently used cell model for PD [17]. We did not use differentiated cells since over 2/3 of studies with this model are without differentiation. In addition, the process of treating cells with other agents could potentially bring uncertainty to the results [17]. The concentration of 500 μM was used because a preexperiment (see Supplementary Material) indicated that 500 μM for 36 h is an intermediate concentration and time regarding cell viability (60–70%). Additionally, this concentration is in accordance with previous studies [[18], [19], [20]] [[18], [19], [20]] [[18], [19], [20]].

In order to maximize the effect of PEMF, we used the setting of square wave, 75Hz, 3 mT for 60 min per day after referring to known studies as follows. One of our previous reports on adipose tissue-derived MSC cells revealed a higher frequency of 75Hz led to superior effect [21]. Another study on primary murine neonatal cardiomyocytes and of sinoatrial node with 75Hz, 0–6 mT PEMF found that 3 mT could maximize the biological effect [22]. On the other hand, a study on PEMF affecting calcium flux designed a variety of stimulation from 1 min to up to 240 min, which found the duration of 60 min maximized the effect of PEMF [23]. Squared pulses was utilized in our research, which was in consistent with our previous basic study on PEMF [24,25], and other clinical studies [10]. For SH-SY5Y cells, 3 mT is the maximum level of PEMF ever used according to previous reports [26,27]. However, this setting is not used before on this specific model and could not be comparable with that in the treatment of PD patients [10]. Currently, there is no applicable model to describe how exactly the PEMF attenuates over the complex tissues to its target cells.

We identified 27 metabolites that were significantly different between the PEMF and control groups. Among them, four amino acids were differentially regulated; l-glutamine and prolyl-gamma-glutamate were upregulated, while cysteinyl-serine and oxidized glutathione were downregulated.

In our study, the expression level of glutamine in the cell model of PD after PEMF intervention was higher than that in the control group. Glutamine drives various critical processes in mammalian cells and is the main excitatory transmitter in the mammalian central nervous system, but excessive glutamine in synapses could cause neurotoxicity [28]. Based on an SH-SY5Y-A53T model of PD, a recent study also pointed out that glutamine levels in a cell model of PD were significantly increased compared with those in control cells [29]. In another study, Shao et al. reported that increased phenylacetyl-glutamine might be a biomarker to assist in diagnosing PD [30]. However, it is also known that glutamine is an important nutrient for cell metabolism [31]. Previous studies have shown that glutamine can suppress MPP^+^-induced neurotoxicity in PC12 cells by inhibiting the PI3K/Akt signaling pathway, thus reducing oxidative stress damage [32]. This evidence suggests that elevated glutamine may play a paradoxical role in the PD model and thus requires further investigation.

Redox dysfunction and neuro-oxidative stress play a pivotal role in the pathogenesis of PD [33]. In this study, we identified oxidized glutathione (GSSG) being downregulated in PEMF treatment group, which means the reduced/oxidized glutathione ratio was increasing. It has been established that lowered glutathione (GSH) metabolism and GSH/GSSG ratio play a principal role in oxidative stress. An increasing number of studies have reported markedly decreased GSH levels and GSH/GSSG ratios in both the brain tissues and blood of PD patients [[34], [35], [36], [37]] [[34], [35], [36], [37]] [[34], [35], [36], [37]]. The results obtained in our study suggest that PEMFs have the potential to reduce redox dysfunction and thus influence the PD cell model.

In recent years, research on antioxidant approaches has achieved great progress. In Sechi et al.’s study, they administered GSH intravenously to untreated PD patients and found a significant symptomatic efficacy [38]. Moreover, Yamamoto et al. found that the glutathione analog YM737, which is more readily transported into cells than GSH, showed better results than GSH itself in a rat model [39]. Another study also demonstrated that dietary supplementation with S-methyl-l-cysteine could enhance the methionine sulfoxide reductase (MSRA) antioxidant system, thus preventing alpha-synuclein-induced abnormalities and alleviating the symptoms of PD [40]. Several other interventions have been attempted thus far, from traditional antioxidant schemes, such as folic acid, vitamin E, C, and β-carotene supplementation, to newer prevention, such as delivering antioxidant molecules by nanoparticles [41]. However, these attempts focused mainly on targeting GSH. Our study, on the other hand, implies that PEMF could potentially target GSSG to regulate GSH/GSSG ratio, which could be a new therapeutic target.

Our study indicated that PEMF intervention could influence fatty acid ester metabolism, including 3-hydroxydecanoyl carnitine, 3-hydroxytetradecanoyl carnitine, (R)-3-hydroxybutyrylcarnitine and acetylcarnitine. Previous studies have demonstrated that treatments targeting fatty acid esters have potential therapeutic potential in PD. Siamak Afshin-Majd’ study assessed the neuroprotective role of acetyl-*l*-carnitine (ALCAR) in a 6-hydroxydopamine (6-OHDA)-treated PD rat model [42]; this demonstrated that ALCAR could decrease neuroinflammation and oxidative stress in the PD model. Another study in 6-OHDA-induced hemiparkinsonian rats also suggested that ALCAR could attenuate microglial activation in addition to preventing neuronal loss and improving memory functions [43]. However, in our study, the acetylcarnitine level in the PEMF intervention group was lower than that in the control group, which suggested that PEMF might play a negative role in the in vitro model of PD by the aforementioned mechanisms. Considering that the role of PEMF in PD studies is limited, more relevant research is required to clarify the mechanism from a metabolic perspective.

We also found that adenosine monophosphate was involved in nucleotide metabolism, which was upregulated following exposure to PEMFs. It has been shown that the disruption of cyclic nucleotide signaling contributes to striatal dysfunction [[44], [45], [46]] [[44], [45], [46]] [[44], [45], [46]]. Our KEGG analysis also showed that PEMF had an effect on the cAMP signaling pathway by regulating adenosine monophosphate. Mizuno et al. reported that increased cyclic adenosine monophosphate (cAMP) could downregulate the expression of NF-κB and iNOS production, resulting in the suppression of neuroinflammation [47]. A variety of studies regarding PD have illustrated that neuroinflammation is involved in the development of PD, and anti-inflammatory therapy strategies have become an attractive option to attenuate PD disease progression [48]. Our data suggest that PEMF could influence inflammatory pathways by regulating adenosine monophosphate, which could be a therapeutic target.

A previous metabolomics study reported that there was an increased level of taurine in 6-hydroxydopamine (6-OHDA)-induced PD rats, while the high expression of taurine was significantly reduced after basic fibroblast growth factor (bFGF) administration on PD [49], which has been considered a potential candidate for PD treatment by promoting the survival of dopamine neurons [50,51]. Thus, the changes in a series of metabolites involved in the upregulation of taurine might be correlated with the neuroprotective role in PD [49], and electromagnetic stimulation could exert such an effect by regulating taurine metabolism.

Compared to the control group, uridine diphosphate (UDP) was another significantly more highly expressed metabolite in the PEMF intervention group. In neuronal SH-SY5Y cells, a previous study found that MPP^+^ stimulation could increase the release of UDP [52]. Conversely, the increase in UDP can enhance the effect of MPP^+^. UDP is an agonist of the P2Y6 receptor (P2Y6R). It was indicated that UDP/P2Y6R could contribute to the progression of oxidative stress and cell death in MPP^+^-treated SH-SY5Y cells [52]. These results suggest that PEMFs could potentially facilitate MPP^+^-mediated injury in a cell model of PD, which made it a double-edged sword; however, to what extent this would affect the application of such treatment still requires further study.

KEGG pathway enrichment analysis showed that the mTOR signaling pathway and PI3K-Akt signaling pathway were affected by PEMF via adenosine monophosphate. According to previous studies, PEMF could activate the mTOR pathway by upregulating the proteins AKT, MAPP kinase, and RRAGA [53]. Accumulating evidence has shown that the mTOR pathway, especially the PI3K/Akt/mTOR signaling pathway, exerts great importance in regulating cell growth, proliferation, and lifespan and is also a central regulator of the autophagy process [54]. Meanwhile, it has been reported that the mTOR pathway has a complex relationship with PD. Recent progress suggests that the induction of autophagy by either mTOR-dependent or mTOR-independent pathways could enhance the degradation of α-synuclein, thus helping develop novel therapeutic targets for patients with PD [55].

The phosphatidylinositol 3-kinase/protein kinase B (PI3K/AKT) signaling pathway has been widely explored in PD. Activation of the PI3K/AKT pathway has been shown to inhibit autophagy by promoting the survival and growth of dopamine neurons [56]. In addition, the PI3K/AKT pathway could also mediate the process of oxidative stress by targeting GSK-3, mTOR, FoxO3a, etc. [57]. Combined with our current research results, PEMFs might primarily exert a protective influence on in vitro models of PD induced by MPP^+^ through multiple mechanisms, thereby expanding opportunities to develop therapeutics to prevent dopaminergic neuron death and promote the degradation of α-synuclein.

Another important question is whether these findings are contributed to PEMF itself since MPP^+^ was used in this study. For normal SH-SY5Y cells, two studies have pointed out that PEMF could defense against oxidative stress, which is in consistent with our findings regarding GSH/GSSG ratio [58,59]. Other reported effects including overexpression of proteins related to high malignancy, drug resistant, or cytoskeleton re-arrangement were not tested in our study [59]. Further studies are still needed on this issue.

There were some limitations to this study. First, the number of samples in this study was limited; further studies with larger sample sizes are warranted to test and verify these findings. Second, in our study, we only investigated the effects of a specific setting of electromagnetic waves (intensity, 3 mT; frequency, 75 Hz; duration, 60 min) on the in vitro model of PD, but the impacts of other parameters cannot be clarified. Also, the impact of this stimulation in vivo is still not clear. Other different settings of stimulation, timing and even orientation of electromagnetic stimulation should be explored both in vitro and in vivo to clarify the optimal frequency, intensity and duration. Third, we did not differentiate SH-SY5Y cells into dopaminergic phenotype and SH-SY5Y was not the only model of PD. The effect of PEMF on other PD cell models should also be tested in the future.

## 5 Conclusion

Collectively, the purpose of this report was to reveal metabolome profiles of the SH-SY5Y cell model of PD under PEMF exposure. Our findings showed that differentially abundant metabolites affected by PEMF were amino acids and peptides, fatty acid esters, nucleotides, etc. These results indicated that PEMF treatment is capable of regulating the metabolism of glutamine, adenosine monophosphate and taurine in MPP + -treated SH-SY5Y cells in the PD model. Additionally, the mTOR, PI3K-Akt and cAMP signaling pathways are influenced by PEMF intervention. Further studies with various parameters of PEMF stimulus are required to verify these findings. In vitro and in vivo functional studies aiming to identify the protective or detrimental effect of these changes are desired.

## Funding

This work was supported by China Postdoctoral Science Foundation (2023M732470), the Post-Doctor Research Project, West China Hospital, Sichuan University (2023HXBH101), and the National Natural Science Foundation of China (82301643).

## Data availability statement

The original data of the current study are available from the corresponding author on reasonable request.

## CRediT authorship contribution statement

**Li-na Zhu:** Writing – review & editing, Writing – original draft, Methodology, Investigation, Funding acquisition. **Deng Chen:** Writing – review & editing, Writing – original draft, Funding acquisition, Formal analysis, Data curation. **Chengqi He:** Writing – review & editing, Validation, Supervision, Project administration, Methodology.

## Declaration of competing interest

The authors declare that they have no known competing financial interests or personal relationships that could have appeared to influence the work reported in this paper.
