# Supplementary material for: Metabolomics comparison of metabolites and functional pathways in the SH-SY5Y cell model of Parkinson's disease under PEMF exposure
Source: Heliyon. 2024 Feb 16;10(4):e26540. doi: 10.1016/j.heliyon.2024.e26540 (PMC10884933; doi:10.1016/j.heliyon.2024.e26540)
Supplement: Multimedia component 1 [file mmc1.docx]

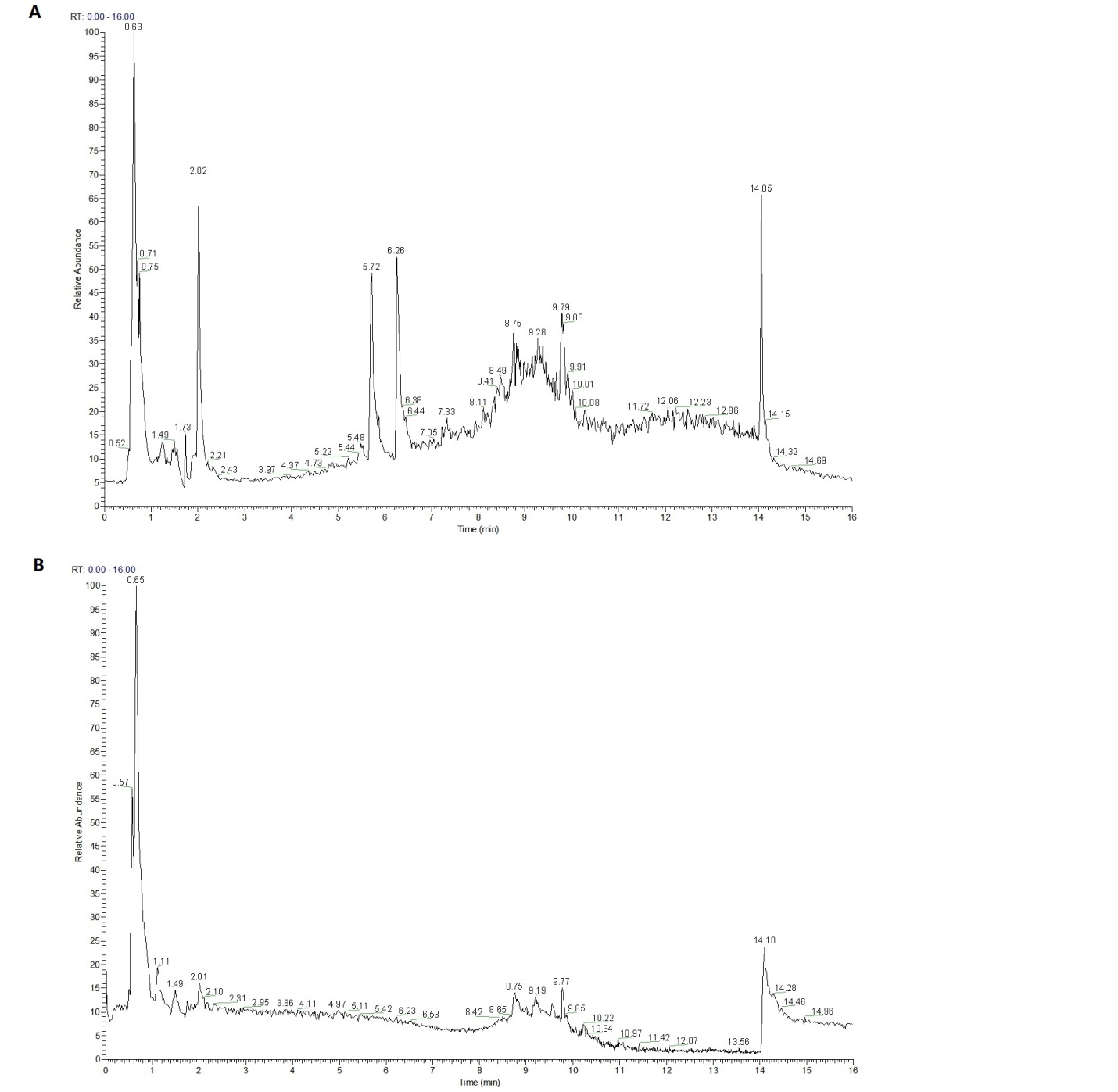


**Fig.1. Total Ion Chromatography (TIC) diagrams.** (A) The positive and (B) negative ion

modes of quality control sample.


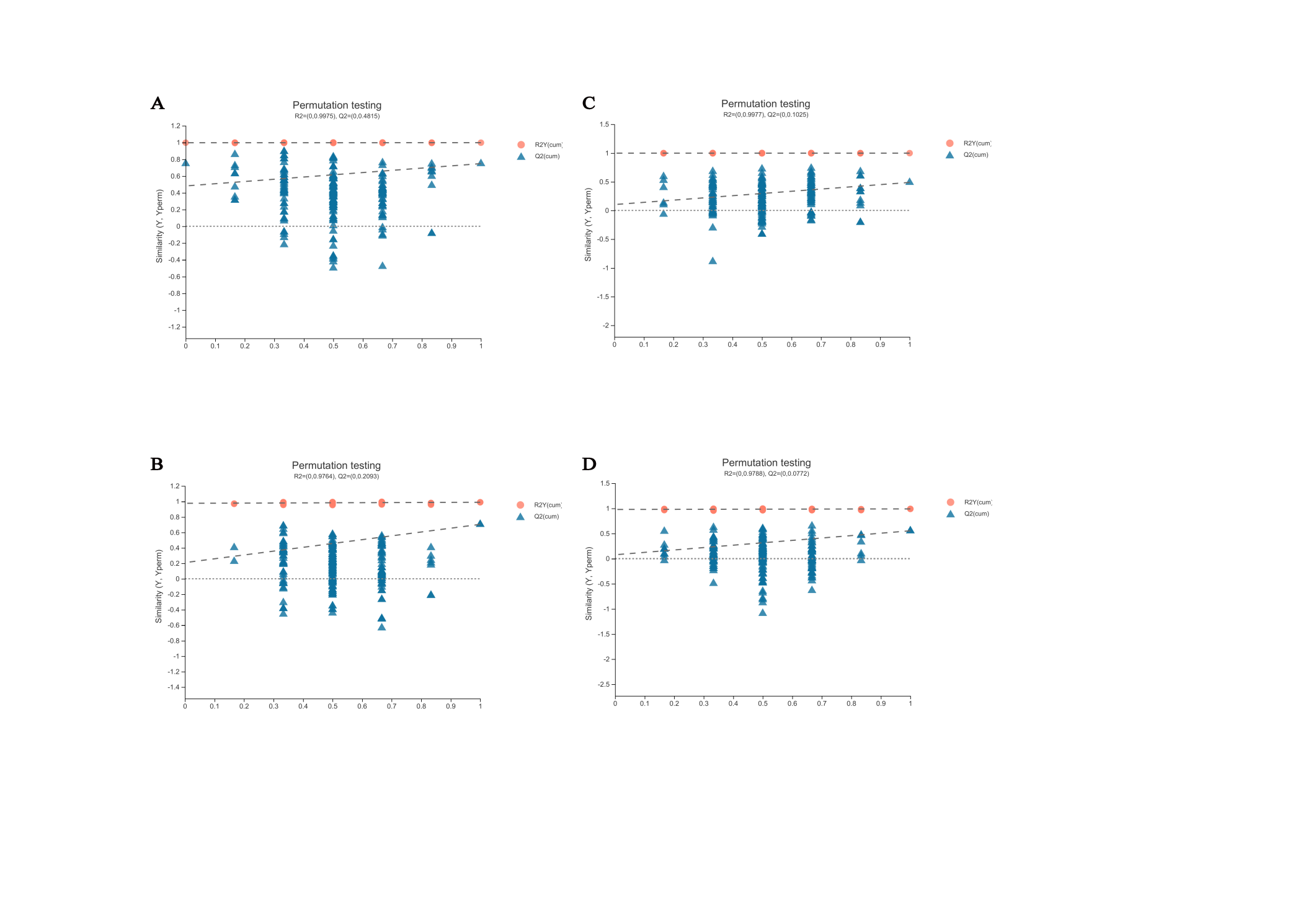


**Fig.2. Response permutation testing (RPT) for model verification.** RPT for partial least squares discriminant analysis (PLS-DA) in the POS (A) and NEG (B) ion modes; RPT for orthogonal partial least squares (OPLS-DA) in the POS (C) and NEG (D) ion modes.


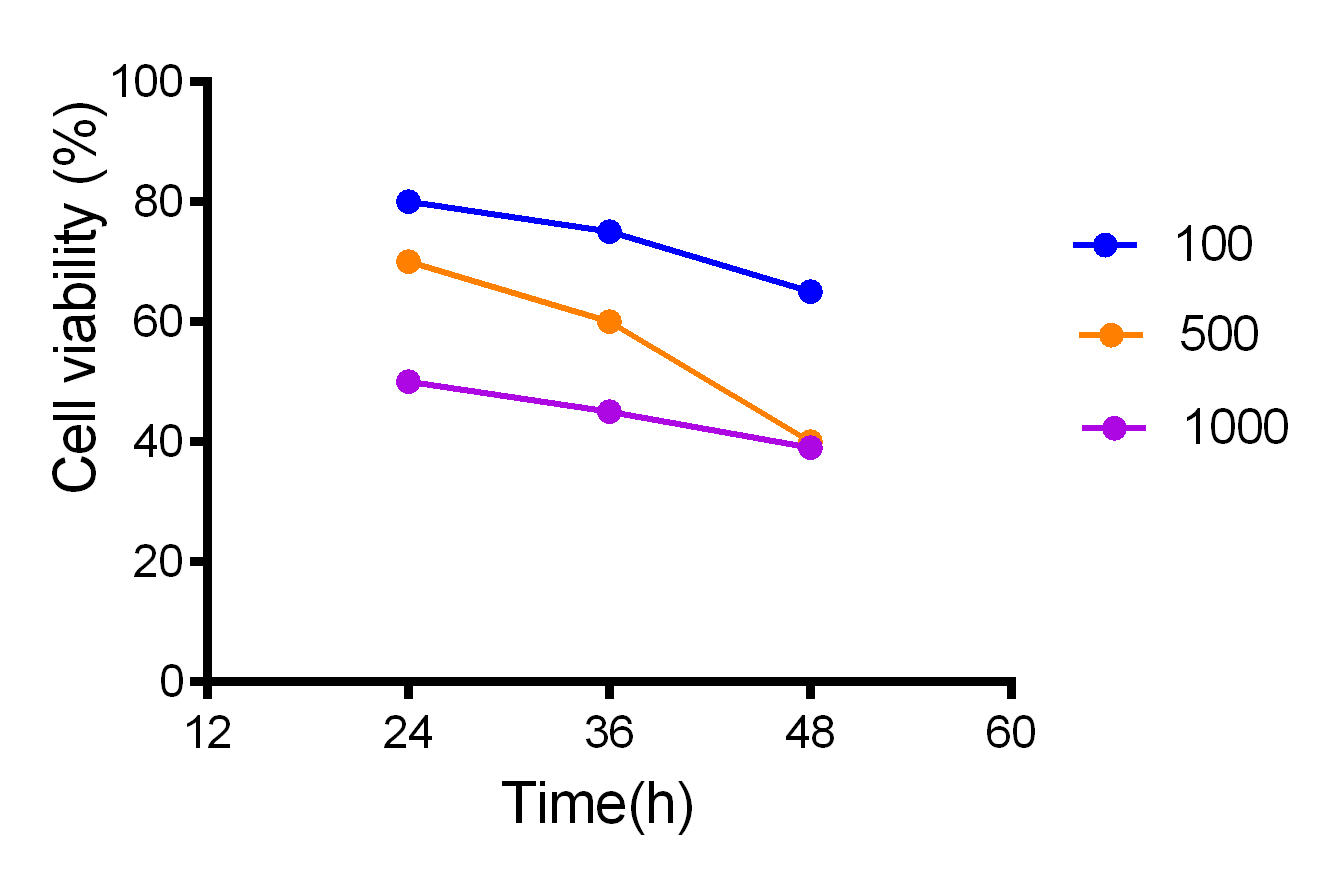


**Fig.3. MTT examination.** Cell viability was monitored by 3-(4,5-Dimethylthiazol-2-yl)-2,5-diphenyltetrazolium bromide (MTT) assay in SH-SY5Y cells treated with various concentrations (100μM, 500μM, 1mM) of MPP^+^ for five time points (12 h, 24 h, 36h, 48h and 60h).

**Table 1.** Differential metabolites identified from the KEGG database

| **pathway ID** | **Description** | **PEMF_vs_Control Metabolites** |
| --- | --- | --- |
| map00010 | Glycolysis / Gluconeogenesis | C00631 |
| map00020 | Citrate cycle (TCA cycle) | C00158 |
| map00030 | Pentose phosphate pathway | C00631 |
| map00120 | Primary bile acid biosynthesis | C00245 |
| map00220 | Arginine biosynthesis | C00064 |
| map00230 | Purine metabolism | C01367;C00064;C00020 |
| map00240 | Pyrimidine metabolism | C00064 |
| map00250 | Alanine, aspartate and glutamate metabolism | C00158;C00064 |
| map00260 | Glycine, serine and threonine metabolism | C00631 |
| map00430 | Taurine and hypotaurine metabolism | C00245 |
| map00480 | Glutathione metabolism | C00127 |
| map00561 | Glycerolipid metabolism | C00631 |
| map00562 | Inositol phosphate metabolism | C01220 |
| map00600 | Sphingolipid metabolism | C00195 |
| map00630 | Glyoxylate and dicarboxylate metabolism | C00158;C00064;C00631 |
| map00750 | Vitamin B6 metabolism | C00064 |
| map00910 | Nitrogen metabolism | C00064 |
| map00920 | Sulfur metabolism | C00245 |
| map00970 | Aminoacyl-tRNA biosynthesis | C00064 |
| map01100 | Metabolic pathways | C00158;C00245;C00631;C00127;C00064;  C01367;C00195;C01220;C00020 |
| map01200 | Carbon metabolism | C00158;C00631 |
| map01210 | 2-Oxocarboxylic acid metabolism | C00158 |
| map01230 | Biosynthesis of amino acids | C00158;C00064;C00631 |
| map01240 | Biosynthesis of cofactors | C00158;C00020;C00064;C00127 |
| map01523 | Antifolate resistance | C00020 |
| map02010 | ABC transporters | C00245;C00064 |
| map04022 | cGMP-PKG signaling pathway | C00020 |
| map04024 | cAMP signaling pathway | C00020 |
| map04068 | FoxO signaling pathway | C00020 |
| map04070 | Phosphatidylinositol signaling system | C01220 |
| map04071 | Sphingolipid signaling pathway | C00195 |
| map04080 | Neuroactive ligand-receptor interaction | C00245 |
| map04150 | mTOR signaling pathway | C00020 |
| map04151 | PI3K-Akt signaling pathway | C00020 |
| map04152 | AMPK signaling pathway | C00020 |
| map04211 | Longevity regulating pathway | C00020 |
| map04216 | Ferroptosis | C00127 |
|  |  |  |
| map04217 | Necroptosis | C00195 |
| map04722 | Neurotrophin signaling pathway | C00195 |
| map04724 | Glutamatergic synapse | C00064 |
| map04727 | GABAergic synapse | C00064 |
| map04740 | Olfactory transduction | C00020 |
| map04742 | Taste transduction | C00158;C00020 |
| map04918 | Thyroid hormone synthesis | C00195 |
| map04922 | Glucagon signaling pathway | C00158;C00631 |
| map04923 | Regulation of lipolysis in adipocytes | C00020 |
| map04924 | Renin secretion | C00020 |
| map04927 | Cortisol synthesis and secretion | C00020 |
| map04928 | Parathyroid hormone synthesis, secretion and action | C00020 |
| map04931 | Insulin resistance | C00195;C02571 |
| map04933 | AGE-RAGE signaling pathway in diabetic complications | C00195 |
| map04934 | Cushing syndrome | C00020 |
| map04964 | Proximal tubule bicarbonate reclamation | C00064 |
| map04974 | Protein digestion and absorption | C00064 |
| map04978 | Mineral absorption | C00064 |
| map05012 | Parkinson disease | C00020 |
| map05022 | Pathways of neurodegeneration - multiple diseases | C00020 |
| map05032 | Morphine addiction | C00020 |
| map05140 | Leishmaniasis | C00195 |
| map05208 | Chemical carcinogenesis - reactive oxygen species | C00127 |
| map05230 | Central carbon metabolism in cancer | C00158;C00064;C00631 |
| map05415 | Diabetic cardiomyopathy | C00195;C00127 |
